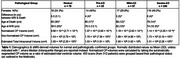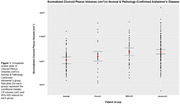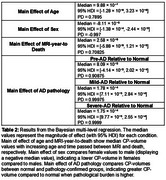# Choroid Plexus Volume in Pathologically Confirmed Alzheimer's Disease

**DOI:** 10.1002/alz70856_101146

**Published:** 2025-12-24

**Authors:** Francis A Fernandes, Marc A Khoury, Adrienne L Atayde, Avyarthana Dey, Nathan W. Churchill, Corinne E. Fischer, David G. Munoz, Tom A. Schweizer

**Affiliations:** ^1^ Keenan Research Centre for Biomedical Science, Li Ka Shing Knowledge Institute, St. Michael's Hospital, Toronto, ON, Canada; ^2^ Institute of Medical Science, Temerty Faculty of Medicine, University of Toronto, Toronto, ON, Canada; ^3^ Keenan Research Centre for Biomedical Science, Li Ka Shing Knowledge Institute, St. Michael's Hospital, Toronto, ON, Canada, Toronto, ON, Canada; ^4^ Department of Physics, Toronto Metropolitan University, Toronto, ON, Canada; ^5^ Temerty Faculty of Medicine, Department of Psychiatry, University of Toronto, Toronto, ON, Canada; ^6^ Temerty Faculty of Medicine, Department of Laboratory Medicine and Pathobiology, University of Toronto, Toronto, ON, Canada; ^7^ Temerty Faculty of Medicine, Division of Neurosurgery, University of Toronto, Toronto, ON, Canada; ^8^ Institute of Biomaterials and Biomedical Engineering, University of Toronto, Toronto, ON, Canada

## Abstract

**Background:**

The choroid plexus (CP), located in the brain's lateral ventricles, plays a vital role in brain homeostasis by clearing cellular/molecular waste. Alzheimer's disease (AD) progression is associated with accumulation of toxic byproducts (hyperphosphorylated Tau & amyloid‐beta) due to impaired cellular/molecular clearance^1^. While CP abnormalities are presumably involved, this has yet to be established. It is thus of substantial clinical interest to identify CP alterations that are indicative of AD pathology burden. We aim to examine CP morphology (volume), as a marker of function, in pathologically‐confirmed AD and hypothesize that CP‐Volume increases with greater AD‐pathology to compensate for the impaired/reduced clearance.

**Methods:**

Structural T1‐weighted magnetic resonance imaging (sMRI) from 312 patients with pathological workup were analyzed from the National Alzheimer's Consortium Centre dataset (Table 1). Patients were grouped based on pathological staging of accumulated tau (Thal staging) and amyloid (Braak staging): Normal (Thal 0‐2, Braak 0‐2), Pre‐AD (Thal 1‐5, Braak 0‐2), Mild‐AD (Thal 1‐5, Braak 3‐4), and Severe‐AD (Thal 1‐5, Braak 5‐6). sMRI underwent automatic segmentation followed by estimation of bilateral CP‐Volumes which were normalized to estimated total intracranial volume. Bayesian multilevel regression was performed by modelling normalized CP‐Volume as a function of pathological status, controlling for age, sex and time between sMRI acquisition and death. Group differences in CP‐Volume were reported in terms of magnitude of effect (i.e.median), probability of direction and 95% highest density interval (HDI), contrasting between normal and pathology‐confirmed groups.

**Results:**

There was a high probability (>0.9) of greater CP‐Volume (Table 2) in the path‐confirmed AD groups compared to normal (Figure 1). The magnitude of effect increased with pathological burden, with mild (1.78×10^−4^ cm^3^, [7.11×10^−5^, 2.84×10^−4^]) and severe‐AD (1.75×10^−4^ cm^3^, [9.77×10^−5^, 2.55×10^−4^]) having the largest effect.

**Conclusions:**

Given existing evidence that impaired clearance is related to AD‐pathology, and CP's involvement in clearance, our findings suggest we can detect morphological changes (via CP‐Volume changes) related to these processes. This warrants further investigation longitudinally and supports the feasibility of sMRI in assessing CP as a correlate of pathological burden.